# Regulatory T-Cell Therapy in Liver Transplantation and Chronic Liver Disease

**DOI:** 10.3389/fimmu.2021.719954

**Published:** 2021-10-14

**Authors:** Angus Hann, Ye H. Oo, M. Thamara P. R. Perera

**Affiliations:** ^1^ The Liver Unit, Queen Elizabeth Hospital Birmingham, Birmingham, United Kingdom; ^2^ Centre for Liver and Gastrointestinal Research and National Institute for Health Research (NIHR) Birmingham Biomedical Research Centre, Institute of Immunology and Immunotherapy, University of Birmingham, Birmingham, United Kingdom; ^3^ Centre for Rare Disease (ERN-Rare Liver Centre), University Hospitals Birmingham NHS Foundation Trust, Birmingham, United Kingdom

**Keywords:** Treg, liver, transplant, cirrhosis, immunity, rejection, cell therapy

## Abstract

The constant exposure of the liver to gut derived foreign antigens has resulted in this organ attaining unique immunological characteristics, however it remains susceptible to immune mediated injury. Our understanding of this type of injury, in both the native and transplanted liver, has improved significantly in recent decades. This includes a greater awareness of the tolerance inducing CD4^+^ CD25^+^ CD127^low^ T-cell lineage with the transcription factor FoxP3, known as regulatory T-Cells (Tregs). These cells comprise 5-10% of CD4^+^ T cells and are known to function as an immunological “braking” mechanism, thereby preventing immune mediated tissue damage. Therapies that aim to increase Treg frequency and function have proved beneficial in the setting of both autoimmune diseases and solid organ transplantations. The safety and efficacy of Treg therapy in liver disease is an area of intense research at present and has huge potential. Due to these cells possessing significant plasticity, and the potential for conversion towards a T-helper 1 (Th1) and 17 (T_h_17) subsets in the hepatic microenvironment, it is pre-requisite to modify the microenvironment to a Treg favourable atmosphere to maintain these cells’ function. In addition, implementation of therapies that effectively increase Treg functional activity in the liver may result in the suppression of immune responses and will hinder those that destroy tumour cells. Thus, fine adjustment is crucial to achieve this immunological balance. This review will describe the hepatic microenvironment with relevance to Treg function, and the role these cells have in both native diseased and transplanted livers.

## Introduction

The tissue damage that occurs in many liver diseases results from immunologically mediated mechanisms (1). This may occur spontaneously, due to the breakdown of self-tolerance, or because of an immune response to a foreign antigen. The major mechanism by which the human body attains self-tolerance is *via* clonal deletion of high affinity self-reactive T-cells in the thymus, however some CD4 T cells escape this process and additional suppressive mechanisms in the periphery are required (2). If both the central and peripheral mechanisms fail, autoimmune disease occurs (3). The liver is unfortunately the site of peripheral tolerance breakdown in several autoimmune liver diseases (AILD). Chronic hepatitis from autoimmune liver diseases can lead to cirrhosis and eventually end-stage liver failure. These conditions include autoimmune hepatitis (AIH), primary biliary cholangitis (PBC), primary sclerosing cholangitis (PSC) and IgG4 mediated hepatitis. In addition, the parenchymal injury that occurs in several chronic viral infections is thought to be the result of an individuals unchecked immune response rather than direct cytotoxic effects of the viruses (4). One of the major challenges in solid organ transplantation is the prevention of immunological rejection. The transplanted liver is susceptible to both cellular and antibody mediated immune injury. Our understanding of the immune mechanisms involved in AILD, chronic inflammatory liver diseases and transplantation has continued to grow over last two decades. An increase in the effectiveness of modern immunosuppressants has translated into better outcomes for these medical conditions and solid organ transplantation (5). The most desirable therapy for autoimmune liver disease and liver transplant recipients is to induce a tolerogenic state, without suppressing other essential protective pathways of the immune system.

A subset of CD4^+^ T-cells proposed to have suppressive abilities was first identified by Nizuzhuka and Sakakura in 1969, subsequently Ghershon and Kondo (1971) demonstrated that thymus derived lymphocytes are required for tolerance induction (6–8). Many decades of further research defined this tolerance inducing T-cell lineage as CD4^+^ CD25^+^ with the transcription factor FoxP3 and known as regulatory T-Cells (Treg) (8, 9). The role of Treg in different autoimmune diseases and autoimmune liver diseases has been the focus of ongoing research in many laboratories including our own, with Treg-directed therapies aimed at augmenting Treg frequency and function undergoing clinical trials in humans (10–15). Treg comprise between 5-10% of CD4^+^ lymphocytes in the systemic circulation and their suppressive effects on effector T-cells comes from several different mechanisms (16–19). Enhancement of Treg function may be beneficial in autoimmune disease, chronic inflammation and transplant tolerance. In contrast, due to an individual’s immune response having a pivotal role in removing tumour cells, Treg inhibition may have anti-oncogenic effects (2).

Tregs have been demonstrated to be present within the liver in different disease states (16). However, the role of intrahepatic Tregs is proving more difficult to delineate and is likely influenced by cells and substances within the hepatic microenvironment (20). If Tregs are considered the suppressive T-cell lineage, T-helper 17 (T_h_17) and T-helper 1 (Th1) cells have the opposing role of being pro-inflammatory or regenerative due to their IL-17, IL-22, IFN-γ, and TNF-α secretion (21). Treg cells have been shown to exhibit plasticity and can be converted to an IL-17 secreting phenotype when exposed to inflammatory environments (22). This phenomenon creates additional challenges for Treg cell therapy to be beneficial rather than harmful. Chronic inflammatory cell infiltrate, fibrous tissue deposition and a distorted microcirculation are changes that occur within the liver parenchyma as cirrhosis develops (23). Therefore, the efficacy, mechanisms and sequalae of Treg therapy is likely different in this microenvironment as Treg mediated induction of effector T-cell anergy may promote oncogenesis. Immunotolerance of a transplanted liver is highly desirable as it would avoid the negative effects of immunosuppressive drugs and the morbidity associated with graft rejection. Treg cell therapy has been demonstrated to be safe in liver transplant recipients, but the efficacy has not been demonstrated consistently (10, 11, 24). This review will describe the current understanding of the liver’s microenvironment and its impact on intrahepatic Treg function in chronic liver disease and liver transplantation.

## Regulatory T-Cells

The process of Treg generation occurs in both the thymus and periphery, resulting in thymus derived Tregs (tTregs) and induced Tregs (iTregs) respectively (25). T cell receptor (TCR) signalling in the thymus appears to mediate the generation of tTregs whereas the generation of iTregs can be mediated by numerous mechanisms including exposure to foreign antigens or the type 1 interferon family (25, 26). The process of tTreg generation from thymocytes is enhanced by CD28 co-stimulation as it increases the intensity and duration of TCR signalling (27). The *de-novo* generation of FoxP3^+^CD4^+^CD25^+^ iTregs from naïve CD4^+^CD25^+^ T cells in the periphery has been demonstrated to occur with persistent exposure to a low dose of a foreign peptides (28) in the presence of TGF-β. A proportion of iTregs are known as T regulatory type 1 cells (Tr1) which are characterised by the co-expression of CD49b and lymphocyte-activation gene 3 (LAG-3), with the ability to secrete high levels of IL-10 and TGF-β (25, 29, 30). In contrast to other types of Tregs which constantly express FoxP3, Tr1 type cells only express FoxP3 temporarily upon activation (29).

As described above, the Treg cell population can be split based on the site of origin but the subpopulations can also be based on phenotype and function as described by Miyara et al. (2009) (31). These authors described three functionally different subpopulation based on FoxP3 and CD45 staining; CD45RA^+^FoxP3^lo^ (resting or naive, rTregs), CD45RA^−^FoxP3^hi^ (activated, aTregs) and cytokine-secreting CD45RA^−^FoxP3^lo^ (cytokine-secreting non-Treg cells) (31). The majority of the aTregs originate from rTregs. Once stimulated the rTregs increase their expression of proliferation marker Ki-67 and FoxP3 (31). The aTregs are terminally differentiated and are short-lived whereas the rTregs have a long lifespan in their resting state. The cytokine-secreting non-Treg cells secrete the largest amount of IL-17 and have the greatest potential to transform into Th-17 cells (31).

In the peripheral blood of healthy humans, Wang et al. demonstrated CD45RA^+^ and CD45RO^+^ comprised 23.9% and 63.6% of CD4^+^ CD25^+^ FoxP3^+^ Tregs (32). These two markers (CD45RA^+^ and CD45RO^+)^ have been reported to be mutually exclusive (33). In liver transplant recipients on calcineurin inhibitor (Tacrolimus) therapy, the frequency of CD45RA^+^FoxP3^lo^ and CD45RA^−^FoxP3^hi^ was decreased in comparison to healthy controls, but CD45^-^FoxP3^lo^ were similar (34). These authors concluded that a limited availability of IL-2 was responsible for Treg cell death. Furthermore, CD45RA^+^ Tregs have been demonstrated to be present in the peripheral circulation at a higher frequency in paediatric liver transplant recipients that have developed graft tolerance in comparison to recipients that have not developed tolerance (35). Data on the frequency of each subset within the liver is more limited, however Zhang et al. reported CD45RA^+^ Tregs to comprise approximately 50% of ICOS^-^ Tregs within livers explanted from children with biliary atresia (19).

**Figure 1:**
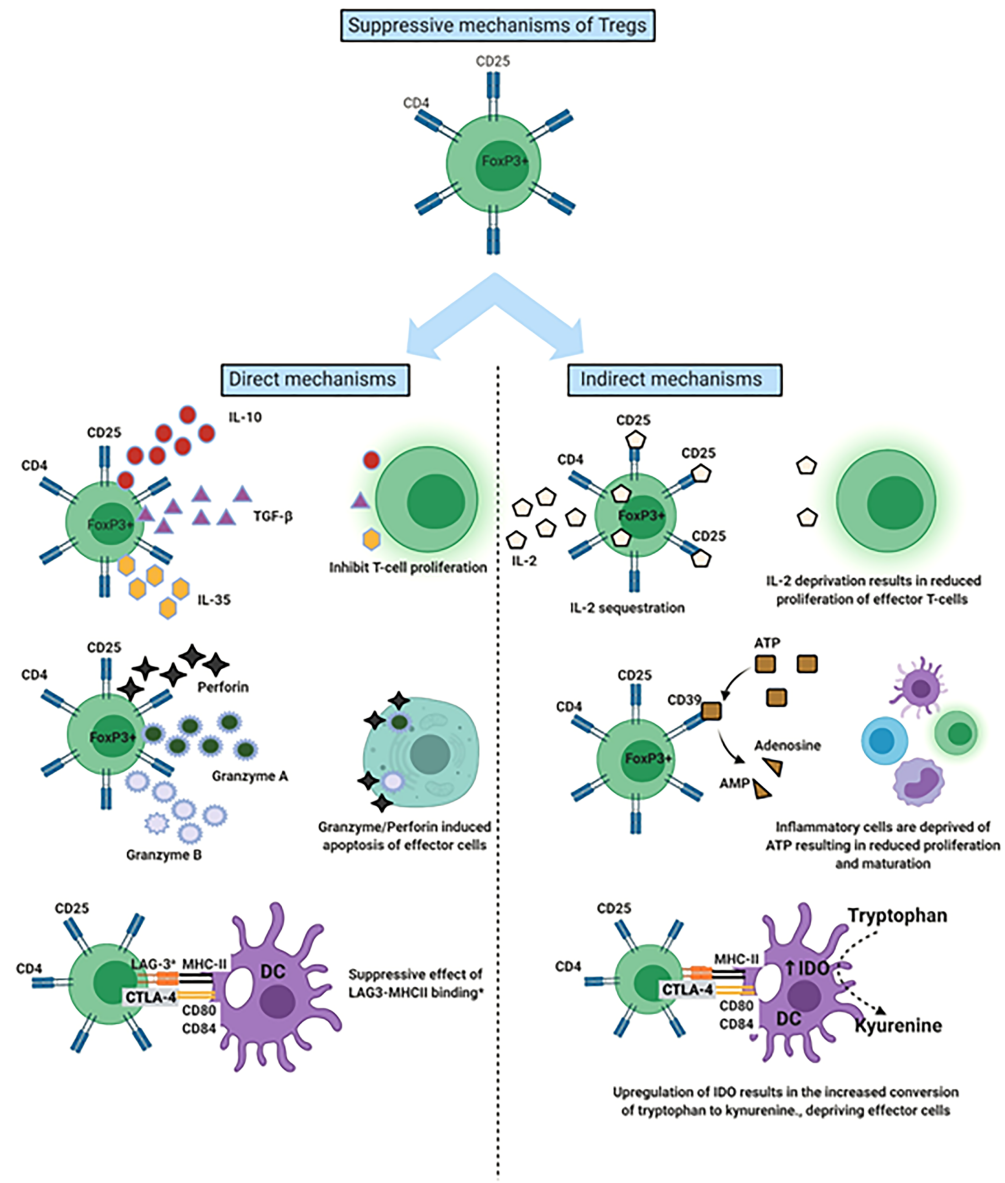
Mechanisms by which Regulatory T cells (Tregs) suppress the immune response. Demonstration of both the direct and indirect mechanisms of Tregs. ^a^LAG-3 involvement most characteristic of T-regulatory type 1 cells. DC, Dendritic cell; LAG-3, Lymphocyte activation gene 3; IDO, Indolamine-2,3-dioxygenase.

To effectively maintain peripheral tissue immune homeostasis, Tregs are required to maintain a stable, anergic and immunosuppressive phenotype (36). The anti-inflammatory and immunosuppressive effects of Tregs have been attributed to both direct and indirect mechanisms (**Figure 1**) (25). Mechanisms include CTLA-4 on Treg leading to trans-endocytosis of CD80/86 molecules on antigen presenting cells, depriving effector T-cells of IL-2 by competitive consumption, depletion of extracellular ATP *via* the release of adenosine through the CD39 molecule on Tregs, secretion of immunosuppressive cytokines (IL-10, IL-35 and TGFβ) and cytotoxic enzymes Granzyme and Perforin to kill T effector cells (25, 37). These functional mechanisms of Tregs can vary depending on given immune scenario, the stimulus and corresponding microenvironment (25, 38, 39). For example, in a murine model of acute liver injury an alleviation of inflammation occurred with adoptive transfer of Tregs and this was associated with increased IL-10 levels within the liver (40, 41).

## The Hepatic Microenvironment

**Figure 2:**
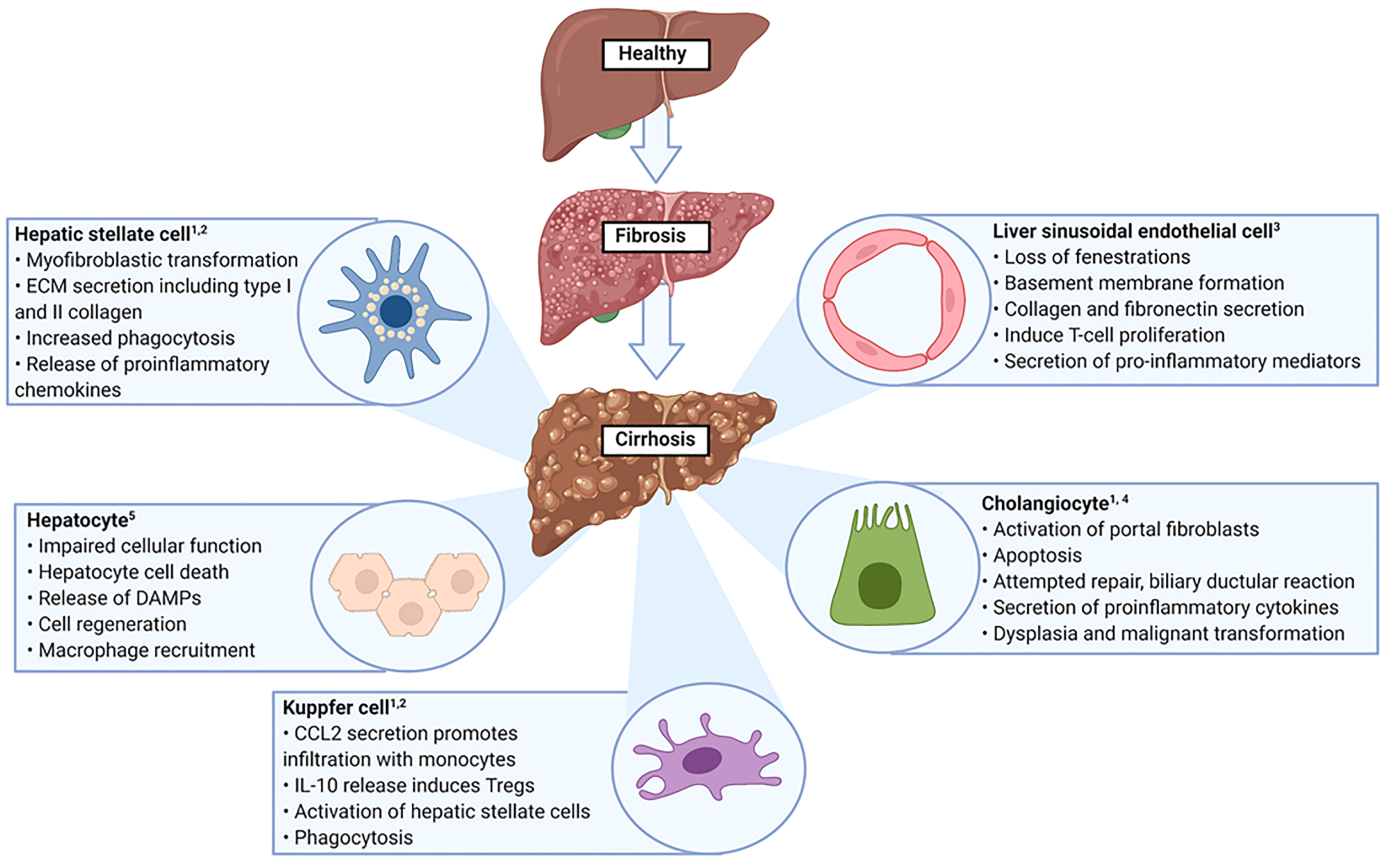
Altered cellular function in liver cirrhosis. ECM, Extracellular matrix; DAMPs, Danger associated molecular patterns. **1.** Yang F et al. Int Immunopharmacol. 2021 Aug 18;99:108051 **2**. Marra F et al. Gastroenterology. 2014 Sep;147(3):577-594. **3**. Poisson J et al. J Hepatol. 2017 Jan;66(1):212-227. **4**. Matsumoto S et al. Liver. 1999 Feb;19(1):32-8 **5**. Kumar S et al. Adv Drug Deliv Rev. 2021 Jul 16:113869.

The liver is the largest internal organ and has a frontline immunological role due to it being positioned to receive gastrointestinal tract derived antigens in the portal venous blood (42, 43). These antigens comprise both pathogenic and non-pathogenic molecules and therefore the liver is required to initiate and amplify an immune response, in addition to displaying a level of tolerance to non-pathogenic organisms (42). The liver microenvironment is comprised by the different cells and molecules that are resident in the liver or transiting through, it is organised to ensure that multidirectional signalling can occur between its different components (44). For example, TGF-β is a strong immunosuppressive cytokine that induces CD4^+^CD25^+^FoxP3^+^ Tregs (45). However, if high levels of IL-6 are also present, this combination (IL-6 and TGF-β) results in generation of IL-17 producing Th17 cells and suppression of Treg induction (45). Therefore, the outcome of an immune response is influenced by the local hepatic microenvironment and understanding how this environment changes in disease states is essential (46). The cellular alterations that take place in liver cirrhosis are summarised in **Figure 2**.

### Blood Supply

The liver is unique in that it has a dual blood supply (42, 47). In health, 70% of the blood volume delivered to the liver arrives *via* the portal vein which represents the intestinal venous outflow, the remaining 30% comes *via* the hepatic artery (47). Aberrations in portal vein flow influence the hepatic artery *via* hepatic arterial buffer response (HABR) (48). This response entails a compensatory increase in hepatic artery flow if portal flow decreases, and vice versa (48). However, this relationship is unidirectional due to the relatively fixed flow in the portal vein which cannot increase to compensate for a fall in arterial flow. The space of Mall surrounds the terminal branches of the portal venules and hepatic arterioles, between the portal tract stroma and the hepatocytes (49, 50). Adenosine accumulation in the space of Mall is thought to occur in the setting of reduced portal flow, resulting in arterial vasodilation. The HABR has significant implications for certain pathological conditions, such as post hepatectomy liver failure and liver transplantation (51).

### Hepatic Microcirculation

The hepatic microcirculation has been defined as the intrahepatic vessels that have an internal diameter ≤300 μm (52). These largely comprise the portal venules, hepatic arterioles, lymphatics, sinusoids and central venules. The immunosurveillance role of the liver is facilitated by the specific arrangement and function of these vessels (42). Both the hepatic arterioles and portal venules deliver blood *via* short side branches into the hepatic sinusoids, the principal site of flow regulation and molecular exchange in the liver (52). The flow of blood in the sinusoids is approximately 50% slower than in capillaries elsewhere in the body, this allows additional time for pathogen identification (42). Liver sinusoidal endothelial cells (LSECs) comprise 15-20% of the cells in the liver and are integral for many functions of the liver, forming a permeable barrier between the sinusoidal lumen and the space of Disse (53). Their fenestrated arrangement, lack of both a diaphragm and basement membrane result in them being the most permeable endothelial cells in the human body (53, 54). LSECs regulate hepatic vascular resistance and blood flow at the sinusoids *via* release of nitric oxide (NO in response to shear stress (54). Kruppel-like factor 2 is an endothelial specific transcription factor that initiates the synthesis and release of NO and other vasodilating substances (53). Antigen processing and presentation is another role of LSECs and they have demonstrated the ability to prime both CD4^+^ and CD8^+^ T cells *in vitro* and *in vivo* using murine models (46, 55). LSECs facilitate leucocyte adhesion *via* the expression of ICAM-1 and Vascular Adhesion Protein-1 (VAP-1) (53). Trans endothelial migration is enhanced under inflammatory conditions by increased expression of ICAM-1, VCAM-1 and CD31. The common lymphatic endothelial and vascular endothelial receptor (CLEVER-1) has been demonstrated to be present on LSECs in normal liver and this receptor promotes trans endothelial migration of CD4^+^ T cells, specifically Tregs (56). On the abluminal side of the LSEC is the space of Disse which is bounded by hepatocytes on one side, and the basal surface of the LSEC on the other (53, 57). It contains extracellular matrix, hepatic stellate cells (HSC) and substances that have migrated from the sinusoidal lumen (57, 58). Laminin proteins (α, β, γ) and reticular collagen type IV are the predominant proteins of the extracellular matrix (ECM) in the space of Disse (57). The composition of the ECM in the space of Disse alters in disease and this change results in activation of HSC (59, 60).

## Tregs and Chronic Liver Disease

**Figure 3:**
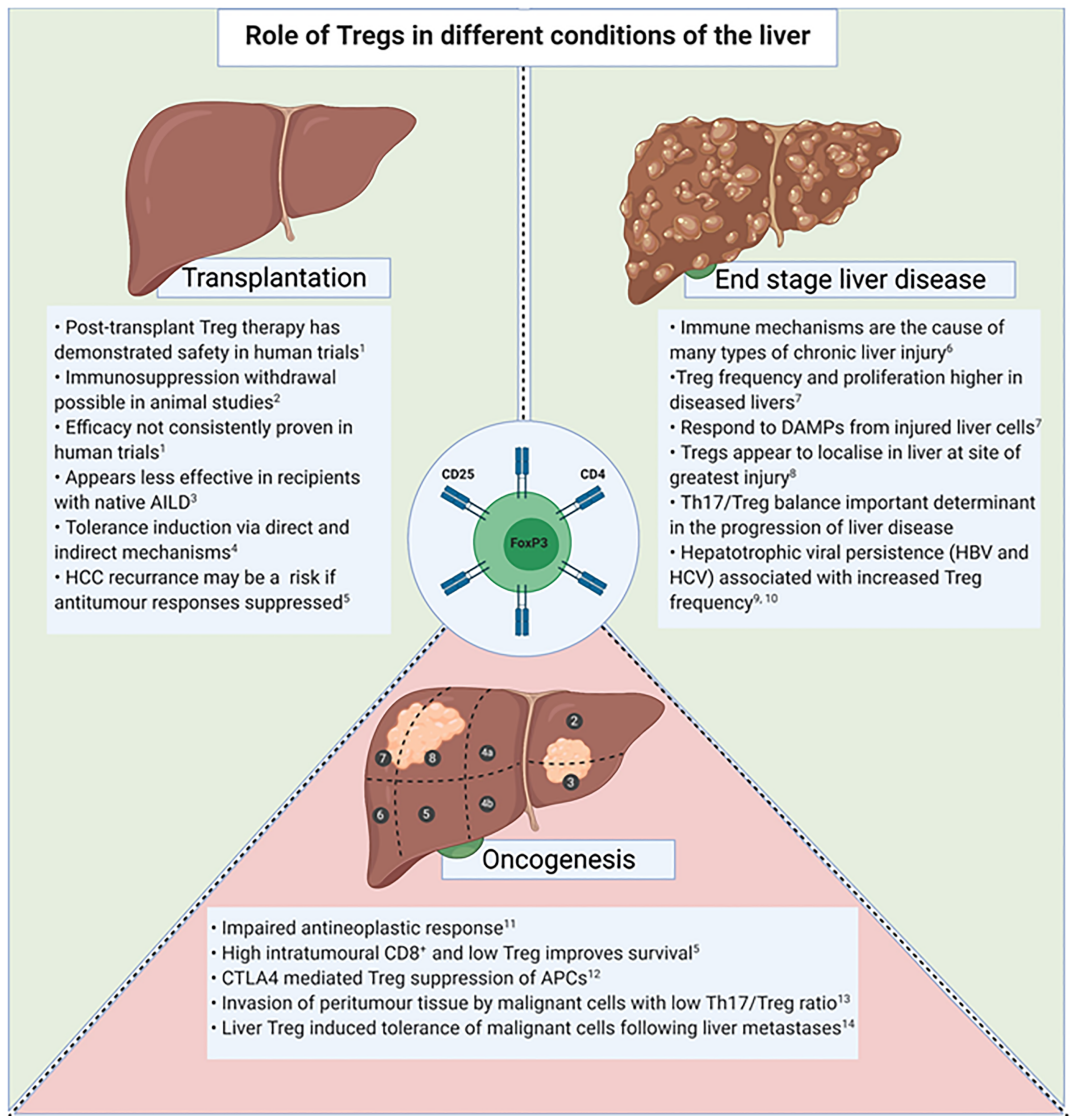
Tregs in the setting of transplantation, end stage liver disease and oncogenesis. AILD, Autoimmune liver disease; DAMPs, Danger associated molecular patterns; APC, Antigen presenting cells; HBV, Hepatitis B Virus; HCV, Hepatitis C Virus. **1.** Sánchez-Fueyo A et al. Am J Transplant. 2020 Apr;20(4):1125-1136. **2.** Bashuda H et al. J Clin Invest. 2005 Jul;115(7):1896-902. **3**. Todo S et al. Hepatology. 2016 Aug;64(2):632-43. **4**. Yu J et al. Liver Transpl. 2021 Feb;27(2):264-280. **5**. Gao Q et al. Journal of clinical oncology.2007;25(18):2586-93. **6**. Eksteen B et al. Seminars in liver disease. 2007;27(4):351-66. **7**. Ikeno Y et al. Frontiers in immunology. 2020;11:584048. **8**. Sasaki M et al. Journal of clinical pathology. 2007;60(10):1102-7. **9**. Tang R et al. Experimental and therapeutic medicine. 2020;20(4):3679-86. **10**. Losikoff PT et al. Virulence. 2012;3(7):610-20. **11**. Shi C et al. OncoTargets and therapy. 2019;12:279-89. **12**. Sachdeva M et al. EXCLI journal. 2020;19:718-33. **13**. Huang Y et al. Journal of gastroenterology and hepatology. 2014;29(4):851-9. **14**. Lee JC et al. Science immunology. 2020;5(52).

Tregs are known to be actively involved in the immune response within both secondary lymphoid tissue and peripheral organs (61). The frequency of Tregs within the liver, known as liver infiltrating Tregs (Tregs_LIT_), is higher in livers from patients with autoimmune, alcoholic and viral related liver diseases in comparison to healthy livers (61). Tregs_LIT_ have demonstrated the ability to suppress T-cell activation in the setting of chronic liver disease, however they have also been associated with reduced matrix metalloproteinases and inhibiting the clearance of fibrosis (61–63). In addition, the suppressive effects Treg_LIT_ have on CD8^+^ T-cell responses has been proposed to contribute to reduced clearance of hepatotrophic viruses (63). Therefore, therapeutic intervention that aims to increase Tregs_LIT_ frequency or function must balance these opposing effects. In chronic liver disease, the impact Tregs have on inflammation, fibrosis, antigen clearance and oncogenesis must be considered (**Figure 3**).

Treg_LIT_ have been shown to localise in close proximity to intrahepatic dendritic cells (DCs) and CD8^+^ T-cells within the diseased liver and the lymph nodes in the hepatic hilum (61). The Tregs_LIT_ leave the hepatic sinusoids through trans-endothelial migration *via* binding with CXCR3 ligands (CXCL9, 10, 11) expressed on the LSEC (61). Our group (2010) demonstrated that in human livers with seronegative hepatitis the Tregs_LIT_ were predominantly in the hepatic parenchyma or lobules, whereas the portal tracts had the highest Treg_LIT_ frequency in PBC, chronic hepatitis C virus hepatitis (HCV) and alcoholic related liver disease (ArLD) cirrhosis (61). The different locations of Tregs_LIT_ in the acutely inflamed livers with unknown aetiology (seronegative hepatitis) suggests that the role and effect of Tregs_LIT_ in acute and chronic liver disease may also differ. Alternatively, it may just reflect the location of the greatest amount of ongoing injury. The latter rationale is supported by the findings of Sasaki et al. (2007) who demonstrated a greater amount of Treg_LIT_ at sites of chronically inflamed portal tracts, in comparison to non-inflamed portal tracts in both PBC and HCV diseased liver (64).

Chronic hepatitis from different aetiologies contributes to the development of fibrosis and subsequent cirrhosis (65). Therefore, therapies that can reduce the chronic inflammatory process may be beneficial in reducing the development of end stage liver disease. In chronically diseased liver, Treg_LIT_ frequencies are increased and a higher proportion are proliferating cells expressed by Ki67 (65, 66). The proposed stimulus is damage associated molecular patterns (DAMPs) such as IL-33 being released from injured hepatocytes (65). In murine experimental models, Treg_LIT_ depletion resulted in the enrichment with pro-fibrotic and inflammatory Ly-6C^high^ CCR2^high^ monocytes and greater collagen deposition in the liver (65).

One of the main driver of inflammation and regeneration within the liver is orchestrated by Th17 which secrete interleukins IL-17A, IL17-F and IL-22 (67). Chronic inflammatory tissue damage occurs with ongoing Th17 stimulation and IL-17 stimulates type 1 collagen release from HSC (67). Liver resident immune cells, especially Kupffer cells, also secrete IL-17 (68). Furthermore, the inflammatory activity of numerous liver diseases has been shown to correlate with IL-17A concentrations (69). The Th17/Treg_LIT_ balance is influenced by the local cytokines and environmental conditions such as hypoxia (68). As an example, IL-10 suppresses Th17 differentiation (68). Chronic hypoxia induces hypoxia inducible factor 1α (HIF-1α), this favours the RORγt transcription factor and Th17 production (68). Mou and colleagues (2019) demonstrated that the frequency of Th17 cells in the peripheral blood was increased in patients with cirrhosis and chronic HBV, in comparison to healthy controls. In addition, haematoxylin and eosin staining of the liver from the group with cirrhosis or chronic HBV demonstrated more fibrosis, inflammatory cell infiltrate and hepatocyte necrosis (70). This suggests that Th17 cells are implicated in the process of liver injury in HBV. Lan et al. (2019) demonstrated that a peripheral blood Treg : Th17 ratio in favour of Th17 cells was an independent predictor of HBV related liver disease progression (71). Therapeutic strategies that alter the balance, by either increasing Tregs or decreasing Th17 cells, may ameliorate the chronic inflammation and subsequent fibrogenesis.

The immune system is also required to clear infectious pathogens and tumour cells from within the liver. Following infection with HBV or HCV, T-cell responses are important in resolving the acute phase of infection and preventing chronic infection (72). Elimination of a particular virus is reliant on a virus specific T-cell response that may be suppressed by Tregs and result in the persistent infection (72). However, much of the liver damage that results from HBV and HCV viral infection may be a result of the host immune response and Tregs may be protective from this aspect (72, 73). Treg frequency in early HBV infection is similar to that of healthy controls, however higher Treg frequency has been demonstrated in those with chronic HBV infection (HBeAg +) (74, 75). Despite a higher frequency of peripheral Tregs in a group of individuals with chronic HBV infection in comparison to healthy controls, Niu et al. demonstrated that the Treg : Th17 ratio was lower in the group with chronic HBV infection (73). Therefore, the increased frequency of deleterious Th17 cells in chronic HBV infection surpasses that of Tregs.

TGF-β levels have been shown to correlate with peripheral Treg frequency in chronic HBV infection and is a proposed mechanism for FoxP3 induction (74). In a recent study utilising a murine model, Tang et al. (2020) demonstrated that HBeAg may directly be responsible for converting naïve CD4 T-cells into Tregs. Taken together, these two mechanisms suggest that HBV manipulates the immune system to create a TGF-β and Treg rich microenvironment that is anti-inflammatory, allowing viral persistence (75). Effective clearance of HCV is associated with intense HLA class I CD8^+^ and HLA class II CD4^+^ T-cell responses to both structural and non-structural viral proteins (76). These responses are not maintained in those who develop chronic infection and the reason for this includes induction of FoxP3^+^ Tregs, CD4^+^ T cell anergy, CD8^+^ T cell exhaustion and impaired DC function. Tregs have been demonstrated to be present at an increased frequency in the blood of humans chronically infected with HCV, in comparison to those that have cleared the virus and healthy controls (76). In addition, Tregs isolated from chronically infected HCV individuals have been shown to supress this virus specific CD8 T cells response (76). Abnormal dendritic cell function as a result of chronic HCV infection is proposed to contribute to the maintenance of the HCV specific Treg response (76).

## Tregs and Chronic Liver Disease Complicated by Oncogenesis

Treg activation may negatively impact antitumour responses *via* various mechanisms, including CTLA-4 upregulation and suppression of antigen presenting cell (APC) activity (77). Hepatocellular carcinoma (HCC) is a known consequence of chronic liver disease. Survival in the setting of HCC has been associated with the extent of tumour immune cell infiltration (78). Working in synergy with innate immune mechanisms and CD4 T-cells, an effective cytotoxic response from CD8^+^ T-cells eliminate cells displaying malignant potential, and therefore Treg mediated suppression may result in favourable tumour conditions (79). A systematic literature review on the prognostic effect Tregs have in cancer found that the presence of tumour infiltrating Tregs in HCC heralds a poorer prognosis (80). Interestingly, a higher density of the pro-inflammatory Th17 cells also was associated with worse overall survival (81). The enhanced oncogenesis that may result with Treg directed therapies is a risk that needs considering (**Figure 3**).

An inflammatory microenvironment within the liver is associated with development of HCC (79). A lower Th17:Treg ratio was found in the tumour compared to the peri-tumour liver by Huang and colleagues (79). These authors suggested that there was an immunosuppressive state within the tumour and this may facilitate immune escape of malignant cells to invade the surrounding peritumour liver (79). Other authors have proposed that Tregs result in further invasion by malignant hepatocytes through a TGF-β mediated transition from an epithelial to a mesenchymal phenotype (82). In a study that examined HCC resection specimens, Gao et al. (2007) demonstrated that a high level activated CD8 cytotoxic T-cells and a low Treg level was associated with both improved overall and disease-free survival (83). These mechanisms, in addition to the fact that Tregs are known to inhibit T-cell responses, suggests that Treg depletion may prove beneficial in the treatment of HCC. Greten et al. (2010) trialled cyclophosphamide in patients with advanced HCC in attempt to deplete Treg frequency and restore anti-tumour immune responses (78). These authors measured alpha fetoprotein (AFP) specific T cell responses before and after Treg depletion. The results demonstrated that an anti-AFP immune response was present in 6/13 patients following Treg depletion with cyclophosphamide (78). Interestingly, metastatic spread of cancers from other organs to the liver has recently been reported to suppress anti-tumour immunity on a systemic basis (84). This tolerance to the malignant cells was demonstrated to be Treg induced and Treg depletion augmented the response to anti-PD-1 therapy (84). The oncogenic nature of chronic liver disease presents additional challenges to the implementation of Treg therapy in this setting.

## Treg and Liver Transplantation

The ability to remove an entire diseased liver and replace it with a non-diseased organ from another individual has prevented a significant amount of premature death, and effectively cured many liver and metabolic diseases. Pharmacological immunosuppression to prevent rejection in the recipient has progressed significantly over the last five decades, however both graft rejection and medication side effects are significant causes of morbidity (5, 85). Therefore, a therapy that can induce tolerance of the graft without immunosuppression has been referred to as a ‘holy grail’ of transplantation by many (24, 86, 87).

### Role of Treg in Maintaining Liver Allograft-Tolerance

Tregs have shown the ability to induce graft tolerance in animal models and have been demonstrated to be present at a higher frequency within liver grafts of spontaneously tolerant humans (88–90).

Immunological rejection of the liver graft occurs when non-self-antigens on the transplanted graft are processed by antigen-presenting cells and recognised by recipient T cells (91). Several different recognised pathways for graft rejection exist (direct, indirect and semidirect), with the main difference being the origin of the APC (92). Primed CD8^+^ T-cells are the main effector cells that respond and induce the damage to the graft (91). An inflammatory microenvironment, such as that which occurs with preservation-reperfusion injury, has been associated with an increased incidence of rejection (93). The induction of MHC II expression on hepatocytes, LSEC and cholangiocytes by inflammation may provide the mechanistic explanation of this phenomenon (91). The proposed immunosuppressive mechanism of Tregs_LIT_ in the setting of transplantation does not differ to that proposed in other disease states with IL-2 deprivation, chemokine secretion and direct inhibition of APCs reported (94).

Human trials of Treg therapy in the organ transplantation arena have yielded mixed results (10–12, 24). The greatest promise was shown by Todo et al. (2016) in which early operational tolerance was achieved in 7 out of 10 subjects following the administration of a Treg-containing cell product that comprised ex-vivo expanded recipient Tregs, that had been co-cultured with irradiated donor lymphocytes (10). This trial was terminated early due to rejection occurring in three subjects that were transplanted for autoimmune liver diseases, however the high rate of operational tolerance achieved at such a short time interval from transplant was viewed as a step forward. The participants in this study had undergone living donor liver transplantation (LDLT) and therefore access to donor tissue antigens was possible ahead of the transplant operation, an option not possible in many countries with predominantly deceased liver transplant programs. During long term follow up of the seven patients with Treg induced tolerance, Todo et al. (2018) demonstrated a variation in peripheral Treg frequency and responsiveness to donor antigens (95). Despite all seven subjects having grafts without evidence of rejection on biopsy, three demonstrated a significant immune response to donor cells on mixed lymphocyte response analysis several years post-transplant (95). Treg frequency in the peripheral blood increased gradually in three, increased then decreased in three, and remained static in one (95). This suggests the changes in the peripheral blood compartment may not accurately reflect cell interactions within the liver graft.

In the ThRIL trial, Sanchez-Fueyo et al. (2020) assessed the donor specific alloimmune response in liver transplant recipients that received an autologous polyclonal Treg infusion post liver transplant (11). This trial was different that by Todo et al. in that it was in the context of deceased donor liver transplantation, as opposed to living donation. Nine liver transplant recipients received polyclonal autologous Treg between 83 to 481 days post-transplant. These authors showed that in the participants that received the higher dose of Tregs (4.5 million/kg), the T-cell response against donor cells was diminished. This effect was not seen to third party cells and therefore suggests tolerance induction specifically to donor antigens. The details and results of subsequent Treg trials in liver transplantation have been extensively compared and contrasted in reviews by others (24, 94). The role of Tregs in liver transplantation is summarised in **Figure 3**.

Maintaining an immunosuppressive phenotype and function is one of the challenges when expanding Tregs for cellular therapy (96). In a murine experimental model, Li et al. demonstrated that Tregs can be converted into IL-17 producing cells and that IL-1β was required for this to occur both *in-vitro* and *in vivo* (97). This conversion has also been demonstrated with human Tregs but IL-2 and IL-15 were thought to be the key to this process (98). However, both rapamycin and cyclosporin A have been shown to inhibit the generation of IL-17 producing cells in a murine model (99). These authors also demonstrated an additional benefit of rapamycin over cyclosporin A was its ability to promote the generation of FoxP3^+^ cells (99). Therefore, Tregs used in the ThRIL trial were expanded in the presence of Rapamycin and IL-2, and this resulted in superior suppressive abilities of the final cell population (96).

Increasing Treg frequency or function within the liver is essential for the tolerance inducing benefits, given the aforementioned mechanisms. In an animal model, Fujiki et al. demonstrated that CD4^+^ CD25^+^ FoxP3^+^ cells had an increased frequency in the liver and spleen of tolerant liver transplant recipients, in comparison to the non-tolerant animals (88). Extraction of these cells showed *in-vitro* suppression of T cell proliferation, and transfer to another animal prolonged the survival of an additional heart graft (88). Intravenous administration of Tregs to achieve tolerance following liver transplantation relies on these cells travelling through the peripheral circulation to reside within the liver. Tracking of intravenously administered Tregs *via* indium labelling was performed by Oo et al. (2019) and these authors demonstrated that only 22-30% of the administered cells are present in the liver at 24 hours post administration in those individuals with a functioning spleen (100). Subsequent assessment at 72 hours demonstrated the proportion of cells within the liver had fallen (100). Functional asplenism resulted in a higher proportion of cells (44.8%) residing in the spleen at 24 hours (100). Once Tregs have migrated out from the hepatic sinusoids, chemokine gradients influence their position and they can reside around the portal tracts or parenchyma depending on the microenvironment (61, 101). Therapeutic efficacy of Tregs in liver transplantation may be enhanced if homing mechanisms can be optimised, enabling these immune modulating cells to exert their direct effect within the graft. Administration of cell therapy directly into the graft prior to implantation may be a novel and worthwhile approach, as the utilisation of ex-situ machine preservation devices are becoming more common. Utilisation of these devices as a platform to deliver Tregs or modulating therapies would avoid the issues of cell homing and minimise the potential for undesirable systemic effects.

### Tregs in Acute Graft Rejection

The assessment of Treg frequency and function post-transplant is confounded by the additional introduction of pharmacological immunosuppression. In addition to suppressing effector cell activity, non-specific immunosuppressant therapies also affect the Treg population (102). Treg frequency in the peripheral blood has been demonstrated to be higher in patients with liver disease awaiting transplantation than healthy controls (103). A reduction in peripheral blood Treg frequency has been observed immediately post-transplant, with a subsequent increase over the first postoperative year but never returning the pre-transplant level (102, 103). The Treg frequency at 12 months post-transplant was reduced in patients that had experienced acute rejection (103). Han et al. (2020) demonstrated that the activated Treg frequency on day 7 was significantly lower in those that developed biopsy proven T-cell mediated rejection (TCMR) (102). A Treg/CD4 frequency of less than 4.7% on post-transplant day 7 was shown to predict biopsy proven TCMR with a sensitivity and specificity of 100%and 91.4% respectively. Furthermore, these authors demonstrated that the expression of the anti-apoptotic molecule Bcl-2 was reduced on Tregs collected on day 7 post-transplant from patients that experienced rejection, in comparison to non rejectors (102). Therefore, apoptotic Treg death may explain the reduced frequency of Tregs in acute rejection. In a study of paediatric liver transplant recipients, the frequency of Tregs was significantly lower and the frequency of Th17 cells significantly higher in blood samples taken in patients experiencing rejection in comparison to non-rejectors and healthy controls (104). The frequency of both Th17 and Tregs was reduced by the initiation of immunosuppression (104). Treg cell-based therapy administered at the onset of acute rejection or in treatment resistant cases have not been trialled in humans but represents an interesting concept.

### Tregs in Chronic Graft Rejection

As time progresses from transplant, acute TCMR becomes less frequent as this most commonly occurs in the first 6 weeks post-transplant (5). However, a chronic form of rejection can substantially contribute to graft dysfunction and loss (105, 106). Chronic rejection is characterised by ≥50% loss of bile ducts and ≥25% loss of arteriole within the portal tracts, perivenular bridging fibrosis and fibro-intimal hyperplasia of the large perihilar arteries (107). The indirect pathway is proposed to be responsible for chronic rejection (106, 107). Wan et al. (2012) demonstrated the dominance of Th2 cytokines, particularly IL-10, in an animal model of chronic rejection. These authors proposed that the Th2 response accelerates the production of alloreactive antibodies (106). Minimal literature exists describing Treg frequency, localisation and function in human liver graft recipients with chronic rejection. In recipients of renal grafts, patients with chronic rejection have a significantly lower peripheral blood frequency of CD25^high^CD4 Tregs than healthy controls and tolerant patients (108). At present, there is no specific therapy for chronic rejection of a liver graft that will reverse the pathological changes. If organ function is significantly compromised, re-transplantation is the only option. The effect of augmenting Treg frequency and function in the setting of chronic rejection remains unknown at present.

## Treg Inducing or Modulating Therapies

An alternate strategy to administering ex-vivo expanded Tregs, in both chronic liver disease and transplantation, is *in vivo* induction. Modulating the native Tregs *via* promoting cellular expansion or increasing their immunosuppressive potency may prove beneficial in numerous different types of liver disease. Administration of Interleukin-2 (IL-2), a cytokine that has been demonstrated to control the development of CD4^+^ T cell subsets including Tregs, is an example of this strategy and has been investigated in both liver disease and transplantation (109). The main role of IL-2 was initially proposed to be the development of effector T-cells; however it was later demonstrated to be the activation and maintenance of Treg cells, especially as low dose IL-2 selectively stimulates Tregs. The diseased liver microenvironment has previously been demonstrated to be deficient in IL-2 (110).

The effectiveness of IL-2 therapy in liver disease has been investigated in animal models and human subjects with autoimmune liver disease. Buitrago-Molina et al. (2021) utilised a murine model of autoimmune hepatitis to investigate the effect of co-administering IL-2 with anti-IL-2, the rationale for including the latter was to reduce the non-Treg cellular impact of IL-2 (111). These authors demonstrate a significant increase in the frequency of Tregs both in the blood and liver compartment following IL-2 administration. In addition, this resulted in a reduction in both the aspartate transaminase and the inflammatory gene profile of the treated animals (111). In a clinical trial that included patients with various autoimmune disease, two with AIH and four with PSC, demonstrated consistent increase in Tregs across the different diseases (15). This study was designed to determine the appropriate dose of IL-2 and its safety, rather than therapeutic efficacy (15). In various non-liver experimental transplant models, IL-2 therapy has been shown to increase graft or recipient Tregs (112, 113). The ability of IL-2 therapy to restore operation tolerance of the liver allograft is currently under investigation in the LITE trial, assessing the ability of low dose IL-2 to allow complete discontinuation of immunosuppressive therapy following liver transplantation. The results of this trial and those of investigators in the United States (NCT02739412) performing a phase II trial are highly anticipated.

## Conclusion

The prospect of manipulating Tregs to protect the liver from immunologically mediated damage in chronic liver disease or transplant setting is being met with excitement in the scientific community. Intense research is being undertaken in many different centres to further understand Treg cell biology and potential therapeutic applications. Due to the numerous different cell populations in the liver, the effect of Tregs is undoubtedly influenced by this microenvironment and crosstalk with other immune cells. Further detailed understanding of Treg biology in chronic liver disease is required before proceeding from early phase trial to Phase II clinical trial. Therapeutic application of Tregs in the setting of liver transplantation has progressed to phase I trials and has yielded mixed results. Safety of therapeutic Tregs has been demonstrated in select transplant recipients, however the efficacy data on ability to reliably withdraw immunosuppression without immune rejection is yet to be demonstrated consistently.

## Author Contributions

AH drafted the first version of the manuscript. YHO and MTPRP reviewed and edited the manuscript. All authors contributed to the article and approved the submitted version.

## Funding

YO would like to acknowledge the funding provided by the Sir Jules Thorn Biomedical Research Charity for his program of research. AH would like to acknowledge the funding received in the form of the Catherine Marie Enright research scholarship from the Royal Australasian College of Surgeons.

## Conflict of Interest

The authors declare that the research was conducted in the absence of any commercial or financial relationships that could be construed as a potential conflict of interest.

## Publisher’s Note

All claims expressed in this article are solely those of the authors and do not necessarily represent those of their affiliated organizations, or those of the publisher, the editors and the reviewers. Any product that may be evaluated in this article, or claim that may be made by its manufacturer, is not guaranteed or endorsed by the publisher.
